# Two's a Crowd: Phenotypic Adjustments and Prophylaxis in *Anticarsia gemmatalis* Larvae Are Triggered by the Presence of Conspecifics

**DOI:** 10.1371/journal.pone.0061582

**Published:** 2013-04-23

**Authors:** Farley W. S. Silva, Daniel L. Viol, Sirlene V. Faria, Eraldo Lima, Fernando H. Valicente, Simon L. Elliot

**Affiliations:** 1 Department of Entomology, Federal University of Viçosa, Viçosa, Brazil; 2 Applied Biology Group, Empresa Brasileira de Pesquisa Agropecuária Maize and Sorghum, Sete Lagoas, Brazil; Imperial College London, United Kingdom

## Abstract

Defence from parasites and pathogens involves a cost. Thus, it is expected that organisms use this only at high population densities, where the risk of pathogen transmission may be high, as proposed by the "density-dependent prophylaxis" (DDP) hypothesis. These predictions have been tested in a wide range of insects, both in comparative and experimental studies. We think it pertinent to consider a continuum between solitarious and gregarious living insects, wherein: (1) solitarious insects are those that are constitutively solitary and do not express any phenotypic plasticity, (2) the middle of the continuum is represented by insects that are subject to fluctuations in local density and show a range of facultative and plastic changes; and (3) constitutively gregarious forms live gregariously and show the gregarious phenotype even in the absence of crowding stimuli. We aimed to chart some of the intermediary continuum with an insect that presents solitarious aspects, but that is subject to fluctuations in density. Thus, *Anticarsia gemmatalis* (Lepidoptera: Noctuidae) larvae reared at higher densities showed changes in coloration, a greater degree of encapsulation, had higher hemocyte densities and were more resistant to *Baculovirus anticarsia*, but not to *Bacillus thuringiensis*. Meanwhile, with increased rearing density there was reduced capsule melanization. Hemocyte density was the only variable that did not vary according to larval phenotype. The observed responses were not a continuous function of larval density, but an all-or-nothing response to the presence of a conspecific. As *A. gemmatalis* is not known for gregarious living, yet shows these density-dependent changes, it thus seems that this plastic phenotypic adjustment may be a broader phenomenon than previously thought.

## Introduction

Parasites represent an important selective force to their hosts [1]. Besides mortality, the costs of this interaction may be expressed in different ways, such as a reduction in reproductive fitness, reduced survival or inhibition of metamorphosis as seen in insects (see [2]–[4]). Both empirical and theoretical studies have examined the higher risk of pathogen transmission for insects experiencing high conspecific densities [5]–[8]. If the infection risk increases with host density, it is expected that hosts subject to strong variations in density evolve mechanisms of resistance that are triggered by these variations, as proposed by the “density-dependent prophylaxis” hypothesis of Wilson and Reeson [9].

Insects that undergo plastic phenotypic changes when at high densities (i.e. density-dependent phase polyphenism) may invest more in prophylactic resistance mechanisms according to a predictable infection risk at moments of crowding [1]. One aspect of these phenotypic adjustments is changes in immune parameters of insects, such as an increase in hemocyte densities, capsule melanization or the encapsulation response [8], [10]. These parameters, and others, can be measured in comparative studies, where species can be characterized according to typical densities and in experimental studies, where the host density is manipulated during the insect's lifetime [11]–[14].

Insects that show phase polyphenism are of particular interest for the second of these approaches. Phase polyphenism is a fairly widespread phenomenon in insects, having been recorded in Lepidoptera, Orthoptera, Coleoptera and Hemiptera [1], [7], [8], [11]; the "phase" seen when individuals live in crowded conditions is known as *gregaria* and is characterized by individuals with darker or more melanized cuticules than individuals of the *solitaria* phase [10]. Phase polyphenism comprises a suite of phenotypic characteristics, including color, morphology, ontogeny, behaviour and disease resistance (e.g. [15]–[18]).

**Figure 1:**
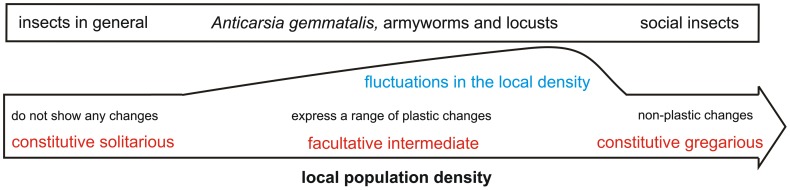
The continuum of phenotypic traits between solitarious and gregarious insects. At the beginning of the continuum are constitutive solitarious insects which present a relatively static local population density (i.e. insects in general), and thus, do not express any phenotypic plasticity. In the middle of the continuum are insects that present fluctuations in the local population density (e.g. *Anticarsia gemmatalis* and armyworms, probably towards to the solitarious end and locusts towards the gregarious end) and will express a suite of facultative and plastic changes in traits. At the end of the continuum are constitutive gregarious insects (e.g. social insects) that present a static local population density and will express a gregarious phenotype regardless of crowding stimuli.

Species that show phase polyphenism, such as armyworms (*Spodoptera exempta* and *S. littoralis*; Lepidoptera: Noctuidae) and locusts (e.g. *Schistocerca gregaria*, *Locusta migratoria* or *Nomadacris septemfasciata*; Orthoptera), are among the most notorious of pests known to man, and phase polyphenism is of critical importance in this; for example, a locust is, at its simplest, just a grasshopper that has phase polyphenism. A key finding with locusts is that the tactile stimuli experienced during the nymphal period are responsible for behavioural phase changes [19]. In a natural setting, these stimuli can serve as good indicators of conspecific densities, leading to adaptive changes in phenotype. Curiously, despite such advances in understanding of the physiological aspects of phase polyphenism, its evolutionary origins seem to be a neglected area. Although we do not tackle this aspect directly here, we consider it pertinent, from pure and applied perspectives, to consider a continuum between solitarious living, at one extreme, and gregarious living at the other (see Fig. 1). At the solitarious end of this continuum are insects that are constitutively solitary and do not express any phenotypic plasticity when exposed to conspecifics; we expect to find the majority of lepidopteran, orthopteran and indeed insect species at this end of the continuum (although in the absence of data, this is merely a prediction). In the middle of the continuum are insects in an ‘intermediary’ stage (such as *Anticarsia gemmatalis*, armyworms and locusts) which are subject to fluctuations in the local density and which show a range of facultative and plastic changes between phase states. At the gregarious end of the continuum are constitutively gregarious forms that live gregariously and show a gregarious phenotype even in the absence of crowding stimuli [20].

Our aim here is to chart some of the intermediary part of the continuum. As a first step, we take an insect with solitarious apects (much as armyworms), but that is subject to fluctuations in density. *Anticarsia gemmatalis* (Lepidoptera: Noctuidae), despite presenting aspects of density-dependent phase polyphenism, is a species which presents low population density and fecundity (if compared to armyworms [21]–[26]), adults lay eggs singly and larvae live non-gregariously [27]. It likely presents the suite of changes as do many other more commonly studied phase polyphenic species, however as it never (to our knowledge) forms swarms, i.e. it does not aggregate and migrate *en masse*, as do locusts, it might express a less evident phenotypic response to density. As phase traits may be differently affected in each species by environmental and genetic factors, its evolutionary processes may also occur differently [28]. As in other polyphenic species, their body color varies from green, when larvae are reared in isolation, to black when they are reared in high densities [27].

*Anticarsia gemmatalis* is one of the most important pests in soybean agrosystems [29], being widely distributed and causing yield losses in all areas where soybean is cultivated [30]. Although it is mainly controlled by chemical insecticides, it is also widely controlled by two entomopathogens: multicapsid nucleopolyhedrovirus (AgMNPV) (a species-specific pathogen) and *Bacillus thuringiensis* subsp. *kurstaki* (specific to Lepidoptera) [29], [31], [32]. Given its importance as an agricultural pest, and the importance of pathogens in its management, its response to variable densities, in particular potential changes in disease resistance, have a heightened importance. It is known that density can affect biological parameters in *A. gemmatalis*, such as larval development and wet and dry weights and lipid and protein contents in adults [27], [33]. In terms of its prophylactic investment in disease resistance, we thus expected this insect to display an intermediary response to density, in line with the DDP hypothesis. To date, no studies have explicitly considered a species with most solitarious aspects (and probably at the solitarious end) at the intermediary region of Sword's continuum [20]. Our results suggest that the prophylactic responses observed in this species are more specifically dependent of the presence or absence of conspecifics rather than a continuous trait.

## Materials and Methods

### Ethics Statement

No specific permits were required for the described studies which were undertaken in the laboratory with a non-endangered or protected species.

### *Anticarsia gemmatalis* Density Treatments

A stock rearing of *Anticarsia gemmatalis* was maintained on artificial diet according to Hoffmann-Campo et al. [34], at 25±5°C, 70±5% relative humidity and 12 h photophase. For the experiments, adults were allowed to oviposit on sheets of sulphite paper and eggs were separated. Hatching occurred within 24 h of oviposition, and larvae were held in 100 ml opaque plastic pots, lidded and with airholes, at four densities: 1, 2, 4 or 8 larvae/pot. They were kept in a climate-controlled chamber (25±1°C, 60±3% relative humidity and 12 h photophase) until further use. This procedure was adopted in four separate assays (below), and insects were used at 10 days post-eclosion, approximately 4th instar. Throughout, only one insect, randomly chosen, was ever used from a given pot, to avoid pseudoreplication.

### Color of Phenotypes

**Figure 2:**
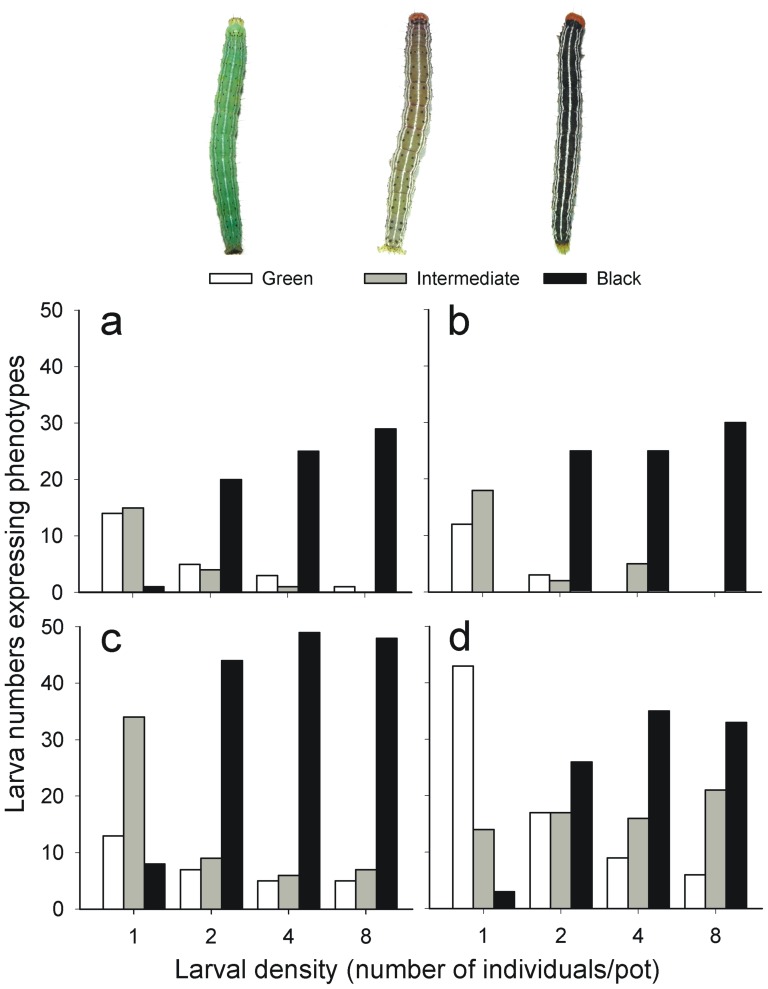
Frequency distribution of *Anticarsia gemmatalis* larvae expressing color phenotypes according to rearing density. Larvae were reared at four densities – 1, 2, 4 or 8 per cup and expressed the phenotypes green, intermediate or black as a result (see statistical tests in text). The green phenotype has an olive-green body color with prominent black spots on it, and head capsules color ranging from green to yellow; the intermediate has black spots arranged on the dorsum and subdorsum, with head capsule color ranging from yellow to orange; the black phenotype has a dark body and a yellow-orange head capsule. Frequency distributions are shown for insects used subsequently in each experiment: **a**) Encapsulation response; **b**) Hemocyte densities; **c**) Susceptibility to *Baculovirus anticarsia*; and **d**) Susceptibility to *Bacillus thuringiensis*.

*Anticarsia gemmatalis* larvae expressed different color phenotypes - green, intermediate or black, according to rearing density. The phenotypes were determined in all four of the experiments that follow, according to coloration of larval head capsules and body. The green phenotype has an olive-green body color with prominent black spots on it, and head capsule colors ranging from green to yellow; the intermediate phenotype has black spots arranged on the dorsum and subdorsum, with head capsule color also yellow-orange; the black phenotype has a dark body and a yellow-orange head capsule (see details in Fig. 2, and [27]).

### Encapsulation Responses

When subjected to invasion by a parasite, one of the immune defences of insects is a triggering of the encapsulation response [35]. To determine if the density of conspecifics affects this response, larvae were submitted to a challenge that simulates the presence of a parasite. Before the experiment, larvae were weighed to ensure homogeneity. Then, a piece of sterile nylon filament (2 mm length and 0,12 mm Ø) was inserted through the first thoracic segment (dorsal region) of 30 larvae/density (two larvae died before the experiment, so n = 118). After 24 h, the larvae were dissected and the nylon filaments were mounted on slides. The slides were photographed (camera Power Shot A640 coupled to light microscope Zeiss Axioskop 40) and two variables were analyzed: capsule area formed around the nylon and capsule melanization, with the aid of IMAGEJ 1.42q software (adapted from [36]). Capsule area was assessed by measuring the area of the layer of cells that formed around the nylon filament. To measure melanization, the pictures were converted to grayscale ranging from 0 to 255, allowing quantification as the mean value of this grayscale for each image (methodology adapted from [37]).

### Hemocyte Densities

Another measure of an insect's immunity is hemocyte densities. As in the experiment above, larvae were weighed before use. Hemolymph extraction was conducted with 30 partially surface-sterilized (70% ethanol) larvae per density, by puncturing a small hole beside the first prolegs. The exuded hemolymph (5 µL) was collected with the aid of a pipette and was held in an Eppendorf tube with 20 µL anticoagulant buffer (98 mM NaOH, 186 mM NaCl, 17 mM Na2 EDTA and 41 mM Citric acid, pH 4.5) plus 12 µL of Giemsa stain. Two aliquots of 8 µL of hemolymph suspension were added in each side of a Neubauer improved chamber (Bright-Line, Precicolor (HBG), Germany) and hemocytes were counted under a microscope. The final value was the mean of the two aliquots, providing the cell numbers per microliter (adapted from [38]).

### Susceptibility to *Baculovirus anticarsia* (AgMNPV)

The virus used in this assay, *Baculovirus anticarsia* or AgMNPV (kindly provided by CNPSo-EMBRAPA), is the principal ingredient of the BaculovirusAAE® bioinsecticide. AgMNPV was chosen as a model in this study firstly because it occurs naturally in populations of *A. gemmatalis*, and secondly because it is used in one of the world's most successful biological control programs [31]. Twenty-four hours before inoculation with AgMNPV, 30 larvae from each density treatment were kept solitarily and starved. Thereafter, square soybean leaf pieces (15×15 mm) were inoculated with 20 µL of virus suspension (6×10^6^ polyhedra/larvae) obtained from the formulated product plus Tween 80 (surfactant diluted to 0,01%) and offered to the larvae to feed for a 24 h period. This viral concentration was chosen as a discriminatory dose in preliminary tests, as it started to kill larvae around the fifth day after inoculation. In the control group, 30 larvae/density were fed with soybean leaf pieces inoculated with 20 µL of distilled water plus Tween 80. The leaf piece is easily consumed by one larva in a single day (ensuring ingestion of a uniform number of viral particles leading to infection); larvae that did not consume the entire leaf piece in this period were excluded from the experiment. Virus-inoculated larvae were kept as above and mortality was assessed daily until death or pupation.

### Susceptibility to *Bacillus thuringiensis* Subsp. *kurstaki* (Bt)

The bacterium used in this assay, *Bacillus thuringiensis* subsp. *kurstaki*, is widely used as the principal ingredient of a lepidopteran bioinsecticide (Dipel® - registered for use against *A. gemmatalis*). This experiment was conducted according to the same methodology as that used for AgMNPV (above). Small soybean leaf pieces were inoculated with 20 µL of Bt suspension (3×10^4^ spores/larva) obtained from the formulated product, and offered to the larvae to feed for a 24 h period. As this pathogen inhibits larval feeding after it begins to act, some larvae did not consume the entire leaf piece during the 24 h period. This concentration was selected in preliminary tests as larvae died between the second and fifth days after inoculation, so we expected to be able to discriminate between rearing treatments.

### Statistical Procedures

Tests of frequency distribution of larval color phenotypes were realized with tests of independence incorporating *G*-tests [17], [39]. All other statistical analyses were performed in R version 2.13.0 [40]. The effects of density, phenotype and weight (the latter used as covariate) on immunity parameters of *A. gemmatalis* were verified using generalized linear models (GLM with normal distribution). All analyses were carried out by fitting a full model and then simplifying it by excluding non-significant terms in order of complexity: interactions, quadratic terms then explanatory variables. The final model was accepted as the simplest model that was not significantly different from the full model or from the previous version of the model. Following the analysis, it was employed in residual analyses to check whether the distribution was the most suitable. Finally, we carried out analyses containing the terms separately, i.e. rearing density (as a categorical variable) or larval phenotype, in order to contrast the factor levels and group them if they were not significantly different. *Anticarsia gemmatalis* larval survival data were analyzed by GLM with censoring (when larvae pupated) and a Weibull distribution, with the aid of the ‘survival’ package [41]. This distribution was chosen by showing the lowest AIC (Akaike's information criterion) value. The models were performed including rearing density, larval phenotype and inoculation status (uninfected or infected by either virus or bacteria) as factors [42].

## Results

### Frequency Distribution of Phenotypes

*Anticarsia gemmatalis* larvae expressed different color phenotypes - green, intermediate or black, according to rearing density – 1, 2, 4 or 8 per pot (see Fig. 2). In all experiments, the frequency of the green phenotype decreased with rearing density, while the frequency of the black phenotype increased with rearing density. The frequency of the intermediate phenotype also decreased with density in all experiments, except in the last (i.e., Bt resistance), where it remained almost constant. Tests of independence show the frequency of larval color to be associated with rearing density in all four experiments [Values of *G* for: (a) encapsulation response, (b) hemocyte densities, (c) virus resistance and (d) Bt resistance experiments are 80.43, 104.51, 78.66 and 79.77, respectively, all greater than χ^2^_0.001__[6]_ = 22.46 for *P*<0.001]. Furthermore, when density 1 is compared with the remainder (densities 2, 4 and 8 pooled), the test of independence shows the larval phenotype to depend, more specifically, upon the presence or absence of conspecifics [Values of *G* for: (a) encapsulation response, (b) hemocyte densities, (c) virus resistance and (d) Bt resistance experiments are 69.89, 90.30, 77.28 and 71.65, all greater than χ^2^_0.001__[2]_ = 13.82 for *P*<0.001]. Tests of independence comparing densities 2, 4 and 8 were not significant (Values of *G* for: (a) encapsulation response, (b) hemocyte densities, (c) virus resistance and (d) Bt resistance experiments are 10.53, 14.20, 1.38 and 8.11, respectively, all lower than χ^2^_0.001__[4]_ = 18.46 for *P*<0.001).

### Encapsulation Responses

**Figure 3:**
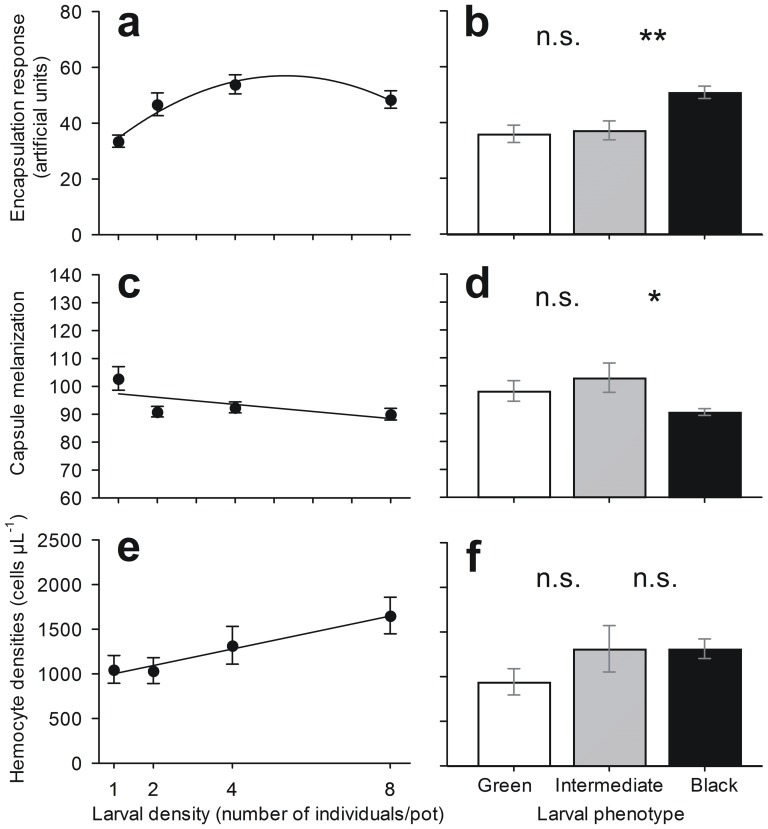
Immune response of *Anticarsia gemmatalis* larvae according to rearing density and resultant phenotype. Larvae were reared at four densities, 1, 2, 4 or 8 per cup, and expressed the phenotypes green, intermediate or black as a result (see Fig. 2); data are shown as responses to these two variables. To assess encapsulation responses and capsule melanization, a piece of sterile nylon filament was inserted through the first thoracic segment of 30 larvae/density, and after 24 h, the nylon filaments were mounted on slides where the two immune defense were assessed with aid of software. **a**) Encapsulation increases with density; shown is the curve of best model fit. The simplest model shows that encapsulation is lower in solitarious larvae than in those in the presence of conspecifics (means±SE). **b**) Less encapsulation occurred in green and intermediate than in black phenotypes. **c**) Capsule melanization decreases with density; shown is the curve of best model fit. The simplest model shows that capsule melanization is higher in solitarious larvae than in those in the presence of conspecifics (means±SE). **d**) More encapsulation occured in green and intermediate than in black phenotypes. To count the cell numbers, a hemolymph aliquot was extracted from each larva (30 larvae/density), placed in a Neubauer improved chamber, and counted under a microscope. **e**) Hemocyte densities increase with density; shown is the curve of best model fit. The simplest model shows that hemocyte densities is a result of the rearing density whereas there was difference between densities 8 and the remaining (means±SE). **f**) Hemocyte densities did not vary with the larval phenotype. Variables were amalgamated when the mean values were not significantly different (*F*-tests: *P<0.05, **P<0.01, *n.s. = *not significant; see table 1 for details).

The degree of encapsulation of the nylon filament increased quadratically with larval density (Table 1; Fig. 3a), implying a threshold density above which encapsulation no longer increases. Note that larval weight did not vary with treatment (*F*_[1,116]_ = 1.619, *P* = 0.206), so it was excluded from the model. To verify if the overall response was principally a function of density *per se*, or the presence or absence of conspecifics, we analyzed density as a categorical variable. The response was observed when we compared the two lowest densities (1 versus 2 larvae/pot) but was not when we analyzed densities in which larvae were in the presence of conspecifics (2, 4 and 8 individuals) versus that in which they were alone (1 individual) (Table 1; Fig. 3a). Besides density, we analyzed the encapsulation response associated with larval phenotype (i.e., phenotype as the independent variable). Both the complete model (all phenotypes) and the simplified model (green + intermediate versus black phenotype) showed that there was a higher encapsulation response in the black phenotype than in the green and intermediate phenotypes (Table 1; Fig. 3b).

**Table 1:** Analysis of deviance table testing the effects of rearing density (1, 2, 4 or 8 larvae/pot), larval phenotype (green, intermediate or black) and weight (used as a covariate) on (a) encapsulation, (b) capsule melanization and (c) hemocyte densities of *Anticarsia gemmatalis* larvae.

source of variation	*df*	*deviance*	*F*	*P>F*
**a) Encapsulation**				
Density	1	2160	7.008	0.009 **
I(densitŷ2)	1	1805	5.857	0.017 *
Phenotype	2	3458	5.608	0.004 **
Weight	1	499	1.619	0.206 n.s.
Residual	117			
*Contrast among the factor levels*				
density (*1 vs. 2 + 4 + 8*)	1	5851	18.545	0.001 ***
phenotype (*green + intermediate vs. black*)	1	5592	17.601	0.001 ***
**b) Capsule melanization**				
Density	1	1383	6.352	0.013 *
Phenotype	2	1526	3.506	0.033 *
Encapsulation	1	176	0.800	0.373 n.s.
Weight	1	1003	4.607	0.035 *
Residual	117			
*Contrast among the factor levels*				
density (*1 vs. 2+4+8*)	1	3066	13.978	0.001 ***
phenotype (*green + intermediate vs. black*)	1	2611	11.695	0.001 ***
**c) Hemocyte densities**				
Density	1	7355043	7.499	0.007 **
Phenotype	2	2148704	1.095	0.338 n.s.
Weight	1	44735	0.046	0.831 n.s.
Residual	119			
*Contrast among the factor levels*				
density (*1+2+4 vs. 8*)	1	6034700	6.123	0.015 *

To verify if the overall immunological responses were a function of density *per se*, or of contact among larvae, we analyzed the density either as a continuous or categorical explanatory variable (contrasting the factor levels). The factor levels were amalgamated if they were not significantly different. (*P<0.05, **P<0.01,***P<0.001, *n.s.* = not significant).

Another immune parameter assessed in this experiment was the capsule melanization formed around the nylon filament. Larval weight did vary with treatments (*F*_[1,116]_ = 4.607, *P* = 0.035), so it was kept in the model as covariate. In this case, however, capsule melanization decreased with larval crowding (Table 1; Fig. 3c). Interestingly, the trigger for the change appears to be the same: when we analyzed density 1 versus 2 larvae/pot (Table 1), the increased capsule melanization occurred in larvae reared alone (Fig. 3c). When analyzed by larval phenotype, we found that capsule melanization was higher in green and intermediate than in black larvae (Table 1; Fig. 3d). As the capsule melanization degree may be correlated with the capsule size (i.e. if there is a fixed pool of melanin to melanize a larger capsule, perhaps it is spread thinner appearing less melanized), we included encapsulation as a covariate in the capsule melanization model, and it was not a significant term (*F*_[1,116]_ = 0.800, *P* = 0.373).

### Hemocyte Densities

Larvae reared in the presence of conspecifics had higher hemocyte densities than those reared alone. In contrast with the previous assays, this was a result of the rearing density as there was difference between density 8 and the other densities (Table 1; Fig. 3e). Hemocyte densities did not vary according to larval phenotype (Table 1). The larval weight did not vary with treatment (*F*_[1,116]_ = 0.046, *P* = 0.831).

### Susceptibility to *Baculovirus anticarsia*

**Figure 4:**
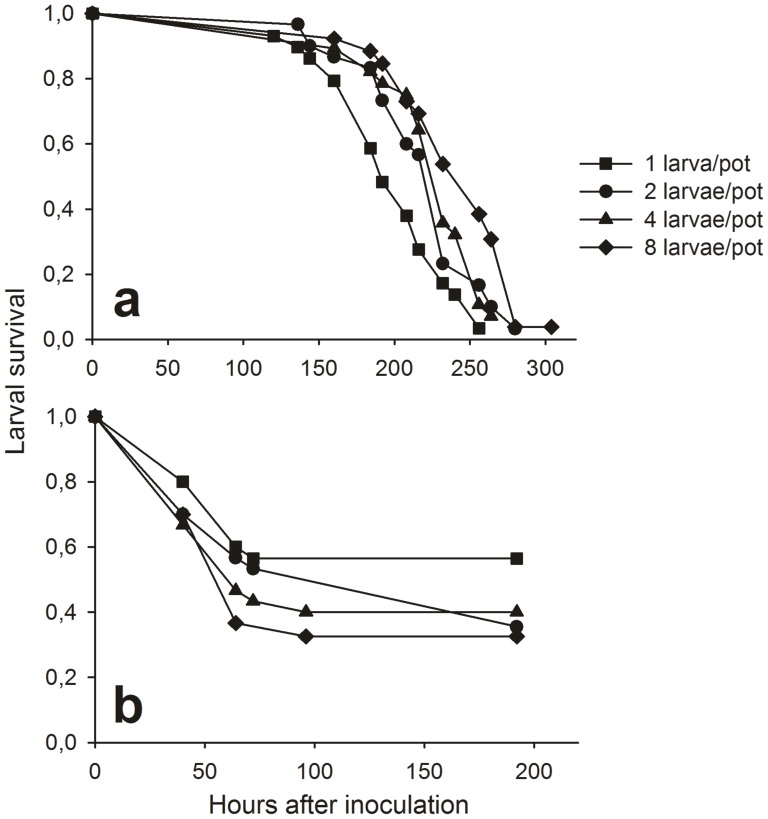
Survival curves of *Anticarsia gemmatalis* larvae. Shown are curves of inoculated larvae with **a**) *Baculovirus anticarsia* or **b**) *Bacillus thuringiensis*. Larvae were reared at four densities – 1, 2, 4 or 8 per cup. Insects of the *Baculovirus* -treatment group were inoculated with 20 µL of suspension (6×10^6^ polyhedra/larvae), while those of the control treatment were inoculated with 20 µL of distilled water. Insects of the *Bt-*treatment group were inoculated with 20 µL of suspension (3×10^4^ spores/larvae), while those of control treatment were inoculated with 20 µL of distilled water. Larval mortality was assessed daily, and the larvae that pupated were included in analysis as censored data. Survival analyses are presented in the text.

Throughout, comparisons between control and infection treatments were highly significant (χ^2^ = 37.51, df = 1, *P*<0.001), with uninfected and infected larvae surviving for mean times of 261±2 (mean±SE) and 221±4 hours, respectively. Larvae of the uninoculated control did not present signs of infection and did not die from virosis, the majority pupating before the experiment end. First, the analysis was performed to check the difference of survival between uninoculated and inoculated larvae. After that, the analysis of density effect was performed including only data of inoculated larvae. Death hazard of inoculated larvae decreased with rearing density (χ^2^ = 37.51, df = 1, *P* = 0.003); as shown by the scale parameter lower than 1. Comparisons among pathogen-inoculated treatments showed that the presence of conspecifics during rearing increased larval survival (density 1 versus 2 larvae/pot, χ^2^ = 17.13, *P* = 0.019; 2 versus 4, χ^2^ = 17.13, *P* = 0.811; 2 versus 8, χ^2^ = 17.13, *P* = 0.038). The survival time of inoculated larvae reared alone (1 individual/pot) was 198±7 hours, while those reared in the presence of conspecifics (2, 4 and 8 individuals/pot) survived for mean times of 220±7, 226±7 and 241±8 hours, respectively (Fig. 4a).

When we analyzed survival of inoculated insects by phenotype, larvae that presented the green phenotype (most of the larvae reared alone) survived for less time (199±11 hours) than the intermediate (218±8) and black phenotypes (226±5), although there was no statistically significant difference (χ^2^ = 37.51, df = 2, *P* = 0.866).

### Susceptibility to *Bacillus thuringiensis*

Throughout, comparisons between control and infection treatments were highly significant (χ^2^ = 101.34, df = 1, *P*<0.001), with uninfected and infected larvae surviving for mean times of 184±3 (mean±SE) and 55±5 hours, respectively. Larval survival did not vary with the rearing density (χ^2^ = 101.34, df = 1, *P* = 0.101), so we did not perform comparisons among pathogen treatments (Fig. 4b). The mean survival times of inoculated larvae reared at densities of 1, 2, 4 or 8 individuals/pot were 54±9, 59±10, 53±10 and 55±8 hours, respectively. The survival times were also not statistically different between the larval phenotypes (green: 55±10 hours, intermediate: 57±8 hours and black: 53±7 hours; χ^2^ = 101.34, df = 2, *P* = 0.447).

## Discussion

In accordance with Sword [20], insects can be considered as living on a continuum between solitarious and gregarious lifestyles. It is mostly insects in the intermediary part of this continuum (i.e. insects that exhibit marked phase polyphenism) that have been used to investigate the Density-Dependent Prophylaxis hypothesis, according to which higher densities lead to elevated investment in disease resistance [7], [8], [11], [16]. This hypothesis has received broad empirical support although there are a number of subtleties in the responses [1] and refinements to the hypothesis have recently been proposed [43]. Meanwhile, work on species that sit at the solitarious end of the continuum tends to indicate that increased densities are a stress factor that increase susceptibility, rather than a prophylactic increase in disease resistance [5], [44]. We feel that it is important to study insects that could be considered to tend towards the solitarious end of the intermediary region of the continuum. Hence the importance of the present study with *A. gemmatalis*, since it is not known for forming large aggregations yet presents density-dependent phase polyphenism. We have shown that increased larval densities lead to changes not only in color phenotypes, but also in the prophylactic investment in immunity. Thus, insects reared at higher densities show a greater degree of encapsulation of the nylon filament used to mimic a macroparasite, have higher hemocyte densities and are more resistant to a virus; meanwhile, with increased rearing density there is reduced melanization of the nylon filament, illustrating the complexities of DDP [1]. Of these parameters, hemocyte density is the only one that did not vary according to larval phenotype. As *A. gemmatalis* is not known for its high population densities, it thus seems that this plastic phenotypic adjustment may be more common than previously thought.

We are aware of no studies that have looked at the mechanistic triggers for DDP but our results may shed some light on this aspect. Our observation that *A. gemmatalis* larvae reared at higher densities are more likely to have a black coloration is in line with Fescemyer and Hammond's study of this species [27]. Here, though, we have found that the observed phenotypic and prophylactic responses are a function, not of density *per se*, but of the presence of (and presumably contact between) conspecifics. In some species, the amount of tactile stimulus received during the juvenile stage is thought to trigger cuticular melanization, through to enzymatic reactions, and increased individual defence to pathogens [7]. In the desert locust *S. gregaria* (one model-species used to test this DDP hypothesis, see [8]), increased tactile stimuli, i.e., repeated touching with a paintbrush of the outer face of nymphs' hind femurs, will elicit individual behavioural phase changes and define what phenotype the individual will adopt [19]. In *A. gemmatalis* then, the stimulus that triggers the phenotypic and prophylactic changes is likely to be similar, since the presence of only one conspecific is sufficient to trigger the response. This is most visible as a higher frequency of the darkest phenotype but can also be seen in the encapsulation and capsule melanization responses, since the insects reared alone (density 1) were significantly different in these parameters from those reared at densities 2, 4 and 8, while comparisons among these densities were not significant. This highlights, at least in part, that it is necessary for only one conspecific to trigger the phenotypic and prophylactic responses in this lepidopteran species, giving a new context to DDP hypothesis. This predicts that prophylaxis is a density-dependent factor, but it does not explicitly treat the relation with pathogen resistance being dependent on the presence of conspecifics. Further experimentation will be necessary to confirm (or otherwise) this threshold effect, taking into consideration the full range of stimuli that may be causing it, and will more investigation of the underlying mechanisms.

We found encapsulation to increase when insects were reared with conspecifics. It was also a function of the larval phenotype, with higher encapsulation occurring in the black phenotype than the green or grey phenoytpes. It was interesting, therefore, to find that capsule melanization decreased with density. Melanization is a process mediated by hemocytes and the fat body [45], and only occurs after the formation of a capsule around the invader (in this study, the nylon filament inserted in larvae) by hemocytes [46]. The finding that some immune parameters (in this case capsule melanization) may respond negatively to increased density is in line with previous work. Thus, Cotter et al. [10] showed that crowded and black larvae of *S. littoralis* (a relative of *A. gemmatalis*) had reduced antibacterial activity while other immune parameters (including capsule melanization, curiously) were increased. This probably reflects trade-offs in the immune defense of insects, due to costs of maintaining these defenses. Povey et al. [47] showed that some immune functions, and consequently survival of infected *S. exempta* larvae (again a relative of *A. gemmatalis*), are directly associated with the intake of protein-rich diets, suggesting a cost to defence. Quite why our study species responded to density with reduced capsule melanization, while *S. littoralis* had the opposite response, is a mystery. The two insects are exposed to quite different patterns of selection, however, and we suggest that further study of non-intermediary species that show phenotypic responses to density should show how consistent this pattern is.

As with encapsulation, hemocyte densities also increased according to the larval rearing density, such that those reared under more crowded conditions had increased hemocyte densities compared to those reared at lower densities. This result reinforces the idea that larvae that are constantly in the presence of conspecifics elevate their immunological responses to counter the increased risk of pathogen exposure. This response seems to differ from encapsulation in that it is triggered by density and not only by presence *per se*. All immune parameters assessed in this study are components of the innate immune system of insects [46], however hemocytes are part of the constitutive defence, in contrast to encapsulation and capsule melanization that represent induced defenses. So it is possible that the density-dependent hemocyte response is associated with its mode of action against threats other than that invading the *A. gemmatalis* hemolymph.

Negreiro et al. [48] suggest that the defense of *A. gemmatalis* larvae to *Baculovirus anticarsia* (AgMNPV) may be attributed to higher hemocyte densities in the hemolymph. In support of this, we found that larvae reared in the presence of conspecifics were more resistant to this virus. Previous work has shown a similarly positive relation between larval density and resistance to viruses, both in the field and laboratory [16], [49]. Goulson and Cory [50] found similar results to the present study, where *Mamestra brassicae* larvae reared at densities of 2, 4 and 10 individuals per recipient and infected with MbNPV presented greater survival than those reared alone.

This same response did not occur for *A. gemmatalis* larvae when faced with another kind of pathogen, in this case *Bacillus thuringiensis* (Bt). The increase in population density did not trigger a prophylactic response against this bacterium: larvae reared at all densities and expressing different phenotypes were equally susceptible (note that the concentration of inoculum we used was chosen to be discriminatory). It is probable that immune responses of these larvae are linked to selective pressure that each pathogen has been exercising on its population. AgMNPV occurs naturally in *A. gemmatalis* populations [31], and due to its specificity [51], it likely had presented a selective pressure driving the evolution of plastic prophylactic mechanisms in this lepidopteran species. According to Wilson and Reeson [9], DDP is a phenomenon that occurs in systems in which pathogens are a regular component of the species' ecology, such as AgMNPV-*Anticarsia gemmatalis* larvae. However, Barnes and Siva-Jothy [11] tested the DDP hypothesis in a non-coevolved insect-pathogen system, and found that it occurs even in a host from a population not previously exposed to that pathogen. In the present study there was not this plastic prophylactic adjustment in larvae inoculated with Bt (a non-specific bacterium that infects a wide range of lepidopteran hosts) [32]. Bt is a pathogen rarely found infecting insects in the field [52], [53]; thus, is unlikely to cause natural epizootics, and there is no reason to expect a density-dependent increase in infection risk. Furthermore, the mechanism for Bt infection is very specific, so it is unlikely that selection pressure from another pathogen (including other bacteria) would affect susceptibility to it.

We feel that it is important to extend the research that has been done on intermediary Lepidoptera and Orthoptera to other insects to see the extent to which the findings on density-dependent changes in phenotype apply to other insects, in particular agricultural pests. The results we present here concern phenotypic plasticity and prophylactic responses in a major pest insect of a major crop plant. They also concern one of the major biological control programs in the world: AgMNPV is one the most extensively used control agents against *A. gemmatalis* in soybean fields [31], and our results suggest that its use at moments of high larval density could mean its failure as a control agent under such circumstances. In screening biological control agents for efficacy, the density of the target pest is rarely if ever considered. However, as pointed out it is not only the density *per se* the key factor in mediating phase change, but rather the presence of conspecifics. Thus, the abundance and resource distribution patterns in the habitat are important ecological factors that might bring individual caterpillars into contact with one another, consequently leading to phase change and DDP syndrome. The approach of such factors together, as has been applied for preventative locust management [54], might be broaden to *A. gemmatalis* management as well.

Furthermore, AgMNPV production is carried out through inoculation *in vivo*, so it is possible that density effects could interefere with large-scale virus production. The passage of AgMNPV through *A. gemmatalis* larvae (i.e., horizontal transmission) living at high densities may lead to negative effects on the virus, such as the production and/or release of viral particles [55] or deletion of genes essential for its infectivity [56], whereas at higher larval densities there will be more barriers to viral replication, such as increased hemocyte densities. We suggest, therefore, that the density at which insects are reared in the early phases of biological control programs be considered critically.

We have shown DDP to occur in an insect that is not known for forming large aggregations, suggesting that the phenomenon may be more common than has been shown so far. Furthermore, we have shown that this phenomenon may be dependent on the presence of conspecifics rather than density *per se*. In fact, we found three different outcomes of the presence of conspecifics, in terms of immune parameters; between densities 1 and 2, one parameter increased, one decreased and one was unaffected, a change occurring over the broader range of densities. This suggests an intriguing divergence in the mechanisms underlying these responses. Changes in immune parameters are intimately associated with other phenotypic traits, most notably coloration, so we are actually observing a suite of characteristics changing together, in effect a change in phase states as in locusts and armyworms. Consistent with this, behavioural changes were noticed (F.W.S.S. personal observation); following changes in coloration phenotype, *A. gemmatalis* larvae moved to different locations of the rearing cage in the laboratory according to color. We suspect that *A. gemmatalis* may represent a transitory step in the evolution of phase polyphenism. In locust species, phase polyphenism is an outcome of multiple evolutionary events within the Acrididae [57]. Within Lepidoptera, phase polyphenism seems to be concentrated within (or restricted to) the Noctuidae. The lymantriid gypsy moth (*Lymantria dispar*) also seems to present features of phase polyphenism [58], but the Lymantriidae, Noctuidae and Arctiidae sit together within the superfamily Noctuoidea. The exact relation between these families remains a matter for debate [59] so determining whether lepidopterans with phase polyphenism are mono- or polyphyletic may not be possible at this stage. However, we feel that it would be very interesting to unravel the phylogenetic origins of phase polyphenism in both Lepidoptera and Orthoptera, and we suggest further studies of this phenomenon in groups that do not display the full suite of phenotypic effects seen in some *Spodoptera* species and in locusts.

## References

[1] Wilson K, Cotter SC (2009) Density-dependent prophylaxis in insects. In: Whitman DW, Ananthakrishnan TN, editors. Phenotypic plasticity of insects: mechanisms and consequences Science Publishers Inc. pp. 137–176.

[2] Hu CY, Rio RVM, Medlock J, Haines LR, Nayduch D, et al.. (2008) Infections with immunogenic *Trypanosomes* reduce *Tsetse* reproductive fitness: potential impact of different parasite strains on vector population structure. PLoS Neglected Tropical Diseases 2.. 10.1371/journal.pntd.0000192 PMC2265429 18335067

[3] Dawes EJ, Churcher TS, Zhuang S, Sinden RE, Basanez MG (2009) *Anopheles* mortality is both age- and *Plasmodium*-density dependent: implications for malaria transmission. Malaria Journal 8. 10.1186/1475-2875-8-228 PMC2770541 19822012

[4] Pruijssers AJ, Falabella P, Eum JH, Pennacchio F, Brown MR, et al (2009) Infection by a symbiotic polydnavirus induces wasting and inhibits metamorphosis of the moth *Pseudoplusia includens* . Journal of Experimental Biology 212: 2998–3006. 19717683 10.1242/jeb.030635 PMC2734494

[5] Steinhaus EA (1958) Crowding as a possible stress factor in insect disease. Ecology 39: 503–514.

[6] Anderson RM, May RM (1981) The population-dynamics of micro-parasites and their invertebrate hosts. Philosophical Transactions of the Royal Society of London Series B-Biological Sciences 291: 451–524.

[7] Reeson AF, Wilson K, Gunn A, Hails RS, Goulson D (1998) Baculovirus resistance in the noctuid *Spodoptera exempta* is phenotypically plastic and responds to population density. Proceedings of the Royal Society of London B 265: 1787–1791.

[8] Wilson K, Thomas MB, Blanford S, Doggett M, Simpson SJ, et al (2002) Coping with crowds: Density-dependent disease resistance in desert locusts. Proceedings of the National Academy of Science 99: 5471–5475. 10.1073/pnas.082461999 PMC122793 11960003

[9] Wilson K, Reeson AF (1998) Density-dependent prophylaxis: Evidence from Lepidoptera-baculovirus interactions? Ecological Entomology 23: 100–101.

[10] Cotter SC, Hails RS, Cory JS, Wilson K (2004) Density-dependent prophylaxis and condition-dependent immune function in Lepidopteran larvae: a multivariate approach. Journal of Animal Ecology 73: 283–293.

[11] Barnes AI, Siva-Jothy MT (2000) Density-dependent prophylaxis in the mealworm beetle *Tenebrio molitor* L. (Coleoptera: Tenebrionidae): cuticular melanization is an indicator of investment in immunity. Proceedings of the Royal Society of London Series B-Biological Sciences 267: 177–182. 10.1098/rspb.2000.0984 PMC1690519 10687824

[12] Robb T, Forbes MR, Jamieson IG (2003) Greater cuticular melanism is not associated with greater immunogenic response in adults of the polymorphic mountain stone weta, *Hemideina maori* . Ecological Entomology 28: 738–746.

[13] Wilson K, Knell R, Boots M, Koch-Osborne J (2003) Group living and investment in immune defence: an interspecific analysis. Journal of Animal Ecology 72: 133–143.

[14] Miller GA, Simpson SJ (2010) Isolation from a marching band increases haemocyte density in wild locusts (Chortoicetes terminifera). Ecological Entomology 35: 236–239.

[15] Leonard DE (1968) Effects of density of larvae on biology of gypsy moth *Porthetria dispar* . Entomologia Experimentalis et Applicata 11: 291–304.

[16] Wilson K, Cotter SC, Reeson AF, Pell JK (2001) Melanism and disease resistance in insects. Ecology Letters 4: 637–649.

[17] Elliot SL, Blanford S, Horton CM, Thomas MB (2003) Fever and phenotype: transgenerational effect of disease on desert locust phase state. Ecology Letters 6: 830–836.

[18] Lee KP, Simpson SJ, Raubenheimer D (2004) A comparison of nutrient regulation between solitarious and gregarious phases of the specialist caterpillar, *Spodoptera exempta* (Walker). Journal of Insect Physiology 50: 1171–1180. 15670864 10.1016/j.jinsphys.2004.10.009

[19] Simpson SJ, Despland E, Hagele BF, Dodgson T (2001) Gregarious behavior in desert locusts is evoked by touching their back legs. Proceedings of the National Academy of Sciences of the United States of America 98: 3895–3897. 11274411 10.1073/pnas.071527998 PMC31149

[20] Sword GA (2002) A role for phenotypic plasticity in the evolution of aposematism. Proceedings of the Royal Society of London B 269: 1639–1644. 10.1098/rspb.2002.2060 PMC1691082 12204123

[21] Milano P, Berti Filho E, Parra JRP, Oda ML, Consoli FL (2010) Effects of Adult Feeding on the Reproduction and Longevity of Noctuidae, Crambidae, Tortricidae and Elachistidae Species. Neotropical Entomology 39: 172–180. 20498952 10.1590/s1519-566x2010000200005

[22] Mensah BA, Gatehouse AG (1998) Effect of larval phase and adult diet on fecundity and related traits in *Spodoptera exempta* . Entomologia Experimentalis et Applicata 86: 331–336.

[23] Sadek MM, Anderson P (2007) Modulation of reproductive behaviour of *Spodoptera littoralis* by host and non-host plant leaves. Basic and Applied Ecology 8: 444–452.

[24] Kazimirova M (1996) Influence of larval crowding and mating on lifespan and fecundity of *Mamestra brassicae* (Lepidoptera: Noctuidae). European Journal of Entomology 93: 45–52.

[25] Rose DJW, Dewhrust CF, Page WW (1995) The bionomics of the African armyworm *Spodoptera exempta* in relation to its status as a migrant pest. Integrated Pest Management Reviews 1: 49–64.

[26] McPherson RM, MacRae TC (2009) Evaluation of transgenic soybean exhibiting high expression of a synthetic *Bacillus thuringiensis* cry1A transgene for suppressing lepidopteran population densities and crop Injury. Journal of Economic Entomology 102: 1640–1648. 19736779 10.1603/029.102.0431

[27] Fescemyer HW, Hammond AM (1986) Effect of density and plant age on color phase variation and development of larval velvetbean caterpillar, *Anticarsia gemmatalis* Hübner (Lepidoptera: Noctuidae). Environmental Entomology 15: 784–789.

[28] Simpson SJ, Sword GA (2009) Phase polyphenism in locust: mechanisms, population consequences, adaptative significance and evolution. In: Whitman DW, Ananthakrishnan TN, editors. Phenotypic plasticity of insects: mechanisms and consequences: Science Plublishers Inc. pp. 147–189.

[29] Bobrowski VL, Pasquali G, Bodanese-Zanettini MH, Pinto LMN, Fiuza LM (2002) Characterization of two *Bacillus thuringiensis* isolates from South Brazil and their toxicity against *Anticarsia gemmatalis* (Lepidoptera: Noctuidae). Biological Control 25: 129–135.

[30] Sosa-Gomez DR (2004) Intraspecific variation and population structure of the velvetbean caterpillar, *Anticarsia gemmatalis* Hübner, 1818 (Insecta: Lepidoptera: Noctuidae). Genetics and Molecular Biology 27: 378–384.

[31] Moscardi F (1999) Assessment of the application of baculoviruses for control of Lepidoptera. Annual Review of Entomology 44: 257–289. 10.1146/annurev.ento.44.1.257 15012374

[32] Beron CM, Salerno GL (2006) Characterization of *Bacillus thuringiensis* isolates from Argentina that are potentially useful in insect pest control. Biocontrol 51: 779–794.

[33] Fescemyer HW, Hammond AM (1988) Effect of larval density and plant age on size and biochemical composition of adult migrant moths, *Anticarsia gemmatalis* Hübner (Lepidoptera: Noctuidae). Environmental Entomology 17: 213–219.

[34] Hoffmann-Campo CBH, Oliveira EB, Moscardi F (1985) Criação massal da lagarta da soja (*Anticarsia gemmatalis*).In: EMBRAPA/CNPSo, editor: Londrina, PR. pp. 23.

[35] Cerenius L, Lee BL, Soderhall K (2008) The proPO-system: pros and cons for its role in invertebrate immunity. Trends in Immunology 29: 263–271. 18457993 10.1016/j.it.2008.02.009

[36] Berggren A (2009) Effect of landscape and population variables on immune response in experimentally introduced bush-cricket populations. Landscape Ecology 24: 749–757.

[37] Dubovskii IM, Grizanova EV, Chertkova EA, Slepneva IA, Komarov DA, et al (2010) Generation of reactive oxygen species and activity of antioxidants in hemolymph of the moth larvae *Galleria mellonella* (L.) (Lepidoptera: Piralidae) at development of the process of encapsulation. Journal of Evolutionary Biochemistry and Physiology 46: 35–43. 20297667

[38] Ibrahim AMA, Kim Y (2006) Parasitism by *Cotesia plutellae* alters the hemocyte population and immunological function of the diamondback moth, *Plutella xylostella* . Journal of Insect Physiology 52: 943–950. 16872627 10.1016/j.jinsphys.2006.06.001

[39] Sokal RR, Rohlf FJ (1995) Biometry: the principles and practice of statistics in biological research; Freeman WH, editor. New York. 887 p.

[40] R Development Core Team (2008) R: A Language and Environment for Statistical Computing. Viena, Austria.

[41] Lumley T (2011) survival: Survival analysis, including penalised likelihood. R package version 2: .36–9.

[42] Crawley MJ (2007) The R Book. Chichester: John Wiley & Sons Ltd. 942 p.

[43] Elliot SL, Hart AG (2010) Density-dependent prophylactic immunity reconsidered in the light of host group living and social behavior. Ecology 91: 65–72. 20380197 10.1890/09-0424.1

[44] Lindsey E, Mehta M, Dhulipala V, Oberhauser K, Altizer S (2009) Crowding and disease: effects of host density on response to infection in a butterfly-parasite interaction. Ecological Entomology 34: 551–561.

[45] Wang Q, Liu Y, He HJ, Zhao XF, Wang JX (2010) Immune responses of *Helicoverpa armigera* to different kinds of pathogens. BMC Immunology 11: 1–12. 20196874 10.1186/1471-2172-11-9 PMC2847984

[46] Marmaras VJ, Lampropoulou M (2009) Regulators and signalling in insect haemocyte immunity. Cellular Signalling 21: 186–195. 18790716 10.1016/j.cellsig.2008.08.014

[47] Povey S, Cotter SC, Simpson SJ, Lee KP, Wilson K (2009) Can the protein costs of bacterial resistance be offset by altered feeding behaviour? Journal of Animal Ecology 78: 437–446. 19021780 10.1111/j.1365-2656.2008.01499.x

[48] Negreiro MCC, Carvalho RBR, de Andrade FG, Levy SM, Moscardi F, et al (2009) Citological characterization of the *Anticarsia gemmatalis* (Lepidoptera, Noctuidae) hemocytes in resistant larvae to the virus AgMNPV. Iheringia Serie Zoologia 99: 66–70.

[49] Reeson AF, Wilson K, Cory JS, Hankard P, Weeks JM, et al (2000) Effects of phenotypic plasticity on pathogen transmission in the field in a Lepidoptera-NPV system. Oecologia 124: 373–380. 28308775 10.1007/s004420000397

[50] Goulson D, Cory JS (1995) Responses of *Mamestra brassicae* (Lepidoptera, Noctuidae) to crowding - interactions with disease resistance, color phase and growth. Oecologia 104: 416–423. 28307656 10.1007/BF00341338

[51] Castrol MEB, Souza ML, Bilimoria SL (1999) Host-specific transcription of nucleopolyhedrovirus gene homologues in productive and abortive *Anticarsia gemmatalis* MNPV infections. Archives of Virology 144: 1111–1121. 10446647 10.1007/s007050050573

[52] Porcar M, Caballero P (2000) Molecular and insecticidal characterization of a *Bacillus thuringiensis* strain isolated during a natural epizootic. Journal of Applied Microbiology 89: 309–316. 10971764 10.1046/j.1365-2672.2000.01115.x

[53] Raymond B, Wyres KL, Sheppard SK, Ellis RJ, Bonsal MB (2010) Environmental factors determining the epidemiology and population genetic structure of the *Bacillus cereus* group in the field. PLoS Pathogens 6: 1–13. 10.1371/journal.ppat.1000905 PMC2873914 20502683

[54] Sword GA, Lecoq M, Simpson SJ (2010) Phase polyphenism and preventative locust management. Journal of Insect Physiology 56: 949–957. 20493192 10.1016/j.jinsphys.2010.05.005

[55] Gomi S, Zhou CE, Yih WY, Majima K, Maeda S (1997) Deletion analysis of four of eighteen late gene expression factor gene homologues of the baculovirus, BmNPV. Virology 230: 35–47. 9126260 10.1006/viro.1997.8457

[56] Pan XY, Long G, Wang RR, Hou SW, Wang HY, et al (2007) Deletion of a *Helicoverpa armigera* nucleopolyhedrovirus gene encoding a virion structural protein (ORF 107) increases the budded virion titre and reduces in vivo infectivity. Journal of General Virology 88: 3307–3316. 18024900 10.1099/vir.0.83363-0

[57] Song H, Wenzel JW (2008) Phylogeny of bird-grasshopper subfamily Cyrtacanthacridinae (Orthoptera: Acrididae) and the evolution of locust phase polyphenism. Cladistics 24: 515–542. 10.1111/j.1096-0031.2007.00190.x 34879633

[58] Ponomarev VI, Andreeva EM, Shatalin NV (2009) Effect of group in gypsy moth (*Lymantria dispar*, Lepidoptera, Lymantridae) related to population characteristics and food composition. Zoologichesky Zhurnal 88: 446–453.

[59] Mitchell A, Mitter C, Regier JC (2000) More taxa or more characters revisited: Combining data from nuclear protein-encoding genes for phylogenetic analyses of Noctuoidea (Insecta: Lepidoptera). Systematic Biology 49: 202–224. 12118405

